# Hints on the Diagnosis and Treatment of Abdominal Affections

**Published:** 1846-12

**Authors:** 


"*Hints on the diagnosis and treatment of Abdominal Affections.* By Dr. Griffin.—In the treatment of acute inflammation of the bowels by purgatives, there is danger of the irritation being propagated from the mucous to the serous surfaces, consequently their use as derivatives cannot be taken advantage of, as in affections of the head and chest. The danger Dr. Griffiin points out, and believes that "people do not recover because they are purged, but they are purged because they recover."

Of the use of opium in peritoneal enteritis, Dr. Griffiin entertains the highest opinion, and relates several cases in which very large and frequent doses were given with the happiest results. In one case, first two grains, and then one grain doses were given every two hours, until thirty-two grains had been taken, two or three grain doses having been resorted to on the occurrence of a relapse. In the case of a girl, ten years of age, whose condition had been previously much aggravated by the use of purgatives, and who appeared to be sinking, twenty drops of laudanum were given, and in half an hour a grain of opium. Sound sleep ensued, and the patient, who had seemed almost moribund, was saved; the opiate being continued for some time at longer intervals. To a boy, *æt.* five, in whom peritonitis occurred during the last stage of typhus fever, probably from perforation, grain doses were given with a successful result. Dr. Griffiin does not propose opium as a substitute for general or local bleeding where these can be borne, but as a most useful remedy where this is not the case, or where the disease continues in spite of their institution. The state of the bladder should be carefully watched, as retention of urine is not of unlikely occurrence during the use of full opiates.

Dr. Griffin does not entirely condemn the use of purgatives, even in the early stages of enteritis, but the greatest advantage is to be derived from them when the force of the disease is broken, and we wish to empty the bowels of their contents. This he effects by mild purgatives, combined with henbane, but he would feel disposed to defer doing this till a later period, if there was no injurious distention present. He concludes, and general experience confirms the conclusion, that in the early stages of enteritis, purgatives do not act until the inflammation has been subdued by deple-.ion, or the disease has otherwise subsided; while, as soon as this has been accomplished, the bowels act spontaneously, or by the use of mild purgatives: that if purgatives are exhibited early, and act freely, death may nevertheless ensue, unless the disease is arrested by other remedies: that purgatives may, *per se*, occasion inflammation, or cause its recurrence; and that inflammation of the bowels may be subdued without any evacuation at all, and the bowels may continue confined for three or four days, without any injurious distension. In distinguishing nervous affections of the abdomen from inflammatory, by tenderness on pressure, Dr. Griffin points out the simultaneous existence in the former of tenderness in a corresponding portion of the spinal column; and where this exists there is a state of system scarcely compatible with inflammation. He says:]

In determining the diagnosis of abdominal inflammation, where both pain and tenderness on pressure exist, we should always endeavor to ascertain—1. Whether there be any pain or tenderness on pressure in the corresponding portion of the spinal column: because, if there be, although it may not absolutely decide whether inflammation be present or not, it is quite sufficient to account for both the pain and tenderness, without assuming the existence of any inflammation. 2. Whether their be no tenderness or pain, the soreness of the abdomen be superficial or deep-seated, which may be ascertained with tolerable certainty in all cases, by an examination directed to that end. And whether, if both superficial and deep-seated, as it usually is in peritoneal inflammation, gentle, steady pressure with the flat of the hand can be easier borne than with the points of the fingers. In pain and soreness from affection of the spinal nerves, it commonly can be so borne, while in peritonitis every kind of pressure, and even the weight of the bed-cloths, is very distressing. (And yet one of the best means of distinguishing hysterical tenderness will often be found to be the observation of how the slighest degree of pressure gives rise to the expression of intense suffering, although the countenance does not always corroborate this. The tenderness in these cases is quite cutaneous.—*Rev.*) 3. Whether the boundaries of the pain or soreness extend beyond what the suspected inflammation could produce. Thus, if inflammation of the liver be suspected, and we find the soreness extending to the ileum or groin, or to the opposite side of the abdomen, it is obvious the soreness cannot be attributable to mere disease of that organ. Again, if the whole abdomen be tender to the touch in a case otherwise closely resembling peritonitis, and we find the tenderness is not confined to the abdomen, but extends over the hips and lower extremities, it is obvious we can attach no importance to the abdominal soreness as a sign of inflammation. (This is a most valuable sign from which we have often derived the greatest assistance.—*Rev.*) Finally, it should be recollected that constipation may depend on mere loss of power in the intestinal nerves, as well as on spasm, obstruction, or inflammation, since the treatment in each case must necessarily be modified, or directed by the supposed cause of this symptom.

[The reviewer remarks that]

Of the antiphlogistic powers of opium, Dr. Griffin expresses his high opinion; and states that for many years he has almost entirely relied upon it for the subdual of enteritis and peronitis. He bleeds first, however, in subjects who will bear depletion, employing calomel also when the disease is very intense, and resists opium powerfully—suspending this as soon as the symptoms give way, and giving the opium alone, whereby troublesome salivation is usually prevented. In rheumatic inflammation it is as useful. In acute inflammation of the mucous membranes opium was formerly supposed to be contra-indicated; but the case published by Dr. Stokes and others, of cure of inflammation of the mucous membrane of the bowels with exhausting diarrhœa show the propriety of the practice. Dr. Griffin attributes the evil effect which have followed the use of opium in these inflammatory affections, of its having been given in too small doses, which, in case of mucous phlegmasiæ, suppress the diarrhœa without subduing the inflammation to which this had been acted as a relief. But in these, as well as in serous inflammations, bleeding must be premised where the strength will admit of it, applying at the same time warm poultices to the abdomen. In other cases in which bleeding is out of question, and opium seems powerless, as in bad dysentery, a combination of ipe cacuanha and opium acts sometimes surprisingly. Dr. Griffin alludes to two cases in which he gave three grains of opium with from three two five of ipecacuanha every two hours with the best effect.

[Although opium is contra-indicated in inflammatory affections of the cerebro-spinal system, from its tendency to produce congestion, yet when combined with sufficient doses of tartar-emetic, it allays nervous irritation, quiets the action of the capillaries, and procures sleep. Its effects are wonderful in many cases of puerperal mania, delirium tremens, and the advanced stages of fever. It is to Dr. Graves we are indebted for pointing out the value of this combination.]

Dr. Graves directs four grains of emet. tart. and two drachms of laudanum to be mixed with half a pint of camphor mixture, two table-spoonsful of which are to be given for the dose, and one every half hour afterwards, until the delirium abates, or some signs of drowsiness appear.

[Dr. Griffin believes that the restorative power of opium in exhaustion from hemorrhage, depends principally upon its property of producing congestion of the brain, and thus restoring tension to the cerebral vessels.]—*Braithwaite's Retrospect.*

